# Recent Advances in Information Security

**DOI:** 10.1155/2014/562492

**Published:** 2014-12-30

**Authors:** Fei Yu, Chin-Chen Chang, Muhammad Khurram Khan, Tianjie Cao, Mirjana Ivanovic

**Affiliations:** ^1^Peoples' Friendship University of Russia, Moscow 117198, Russia; ^2^Feng Chia University, Taichung 40724, Taiwan; ^3^King Saud University, Riyadh 92144, Saudi Arabia; ^4^China University of Mining and Technology, Xuzhou 221000, China; ^5^University of Novi Sad, 21000 Novi Sad, Serbia

Recent years have witnessed rapid developments of the information communication technology and the next-generation Internet. We have seen the potential and value for new technologies from a variety of digital networks to various interconnected digital devices, which make it possible to realize the ubiquitous network and society. But, in such environment, copyright infringement behaviors, such as illicit copying, malicious distribution, unauthorized usage, and free sharing of copyright-protected digital contents, will also become a much more common phenomenon. Researchers, content industry engineers, and administrators attempt to resort to the state-of-the-art technologies and ideas to protect valuable digital contents and services assets against attacks and IP piracy in the emerging ubiquitous network.

This special issue aims to bring together related research works in the realm of ubiquitous network and multimedia contents security and further investigates and discusses trusted solutions and secure techniques for ubiquitous network as well as some open issues faced by digital contents, owners/rights, holders/multimedia, and services providers, who dedicate themselves to protect their intellectual property rights and business benefits.

This special issue includes a collection of 38 papers selected from 151 submissions to 17 countries or districts (Australia, Bulgaria, Canada, China, France, Hong Kong, India, Israel, Korea, Macau, Malaysia, Pakistan, Poland, Saudi Arabia, Spain, Taiwan, and USA). All submitted papers followed the same standard (peer-reviewed by at least three independent reviewers) as applied to regular submissions.

In the paper entitled “*Privacy-preserving location-based query using location indexes and parallel searching in distributed networks*” by C. Zhong et al., an efficient location-based query algorithm of protecting the privacy of the user in the distributed networks is given. This algorithm utilizes the location indexes of the users and multiple parallel threads to search and select quickly all the candidate anonymous sets with more users and their location information with more uniform distribution to accelerate the execution of the temporal-spatial anonymous operations and it allows the users to configure their custom-made privacy-preserving location query requests.

In the paper entitled “*Novel image encryption scheme based on Chebyshev polynomial and Duffing map*” by B. Stoyanov and K. Kordov, a novel image encryption algorithm based on dynamical chaotic systems is proposed. The developed encryption scheme combines Chebyshev polynomial based on permutation and substitution and Duffing map based on substitution. A precise security analysis on the novel encryption algorithm is given.

The paper entitled “*On the improvement of Wiener attack on RSA with small private exponent*” by M.-E. Wu et al. proposes a method, called EPF, to estimate the prime factors of an RSA modulus. With EPF, the cost of exhaustive search can further reduce to 2r-6 bits. As a conclusion, the authors would like to point out that with the continuous improvements in computational capability, the security levels are expected to be higher with the assistance of EPF and the security analysis should be considered more carefully.

In the paper entitled “*Identifying network public opinion leaders based on Markov logic networks*” by W. Zhang et al., a network opinion leader recognition method based on relational data was put forward and an opinion leader recognition system integrating public opinion data acquisition module, data characteristic selection, and fusion module as well as opinion leader discovery module based on Markov logic networks was designed.

In the paper entitled “*A coverage and slicing dependencies analysis for seeking software security defects*” by H. He et al. based on Reverse Data Dependence Analysis Model to extract data dependencies between program statements by analyzing the advantages and disadvantages of the more generally applicable CBFL and program structure slicing method, for shortcomings, the authors proposed a new fault localization method CPSS while retaining the advantages.

The paper entitled “*Separable and error-free reversible data hiding in encrypted image with high payload*” by Z. Yin et al. proposed a separable and error-free reversible data-hiding scheme in encrypted image, which significantly outperforms the previous methods in the three aspects of payload, PSNR, and error rate.

In the paper entitled “*Reducing side effects of hiding sensitive itemsets in privacy preserving data mining*” by C.-W. Lin et al., a novel hiding-missing-artificial utility (HMAU) algorithm is proposed to hide sensitive itemsets through transaction deletion. The transaction with the maximal ratio of sensitive to nonsensitive one is thus selected to be entirely deleted.

The paper entitled “*A complete hierarchical key management scheme for heterogeneous wireless sensor networks*” by C.-M. Chen et al. proposes a complete hierarchical key management scheme which only utilizes symmetric cryptographic algorithms and low cost operations for heterogeneous cluster-based WSN. The analysis and experiments demonstrate that the proposed scheme is secure and efficient; thus, it is suitable for heterogeneous cluster-based WSN.

The paper entitled “*Password-only authenticated three-party key exchange with provable security in the standard model*” by J. Nam et al. presents the first three-party PAKE protocol whose security is proven without any idealized assumptions in a model that captures insider attacks. The model that the authors used to prove the security of our protocol allows the adversary to ask   corrupt queries and thus captures insider attacks as well as forward secrecy.

The paper entitled “*A secure and fair joint e-lottery protocol*” by C.-L. Chen et al. presents a novel joint e-lottery protocol using the multisignature and verifiable random function. Having been proved, the new mechanism can achieve the requirements of general electronic lotteries.

In the paper entitled “*Average gait differential image based human recognition*” by J. Chen and J. Liu, an average gait differential image based human recognition method is proposed. The Kernel idea of AGDI is to apply the average of differential image as the feature image and use the two-dimensional principal component analysis to extract features.

The paper entitled “*Dual key speech encryption algorithm based underdetermined BSS*” H. Zhao et al. presents a new dual key encryption scheme based on the underdetermined BSS problem. The proposed algorithm for speech signals encryption can resist traditional attacks against the encryption system and, owing to approximate calculation, decryption becomes faster and more accurate.

The paper entitled “*High capacity reversible watermarking for audio by histogram shifting and predicted error expansion*” by F. Wang et al. presents a novel reversible audio watermarking algorithm based on improved prediction error expansion and histogram shifting. Experiments show that this algorithm improves the SNR of embedded audio signals and embedding capacity, drastically reduces location map bits length, and enhances capacity control capability.

The paper entitled “*A secure RFID authentication protocol adopting error correction code*” by C.-M. Chen et al. presents a lightweight mutual authentication protocol adopting error correction code for RFID. Compared with other lightweight protocols, the proposed protocol provides stronger resistance to tracing attacks, compromising attacks, and replay attacks.

The paper entitled “*Date attachable offline electronic cash scheme*” by C.-I Fan et al. proposes a provably secure and efficient offline e-cash scheme with date attachability based on the blind signature technique, where expiration date and deposit date can be embedded in an e-cash simultaneously.

The paper entitled “*Efficient and provable secure pairing-free security-mediated identity-based identification schemes*” by J.-J. Chin et al. proposed two SM-IBI schemes that have an instant revocation feature and are very efficient. The authors' schemes outperform the only pairing-based SM-IBI currently known and are provably secure in the random oracle model against both passive and active/concurrent attackers.

In The paper entitled “*A provably secure revocable ID-based authenticated group key exchange protocol with identifying malicious participants*” T.-Y. Wu et al. have fused the Tseng-Tsai R-IDPKS system and a noninteractive confirmed computation technique to propose the first RID-AGKE protocol with identifying malicious participants. The framework and security notions for RID-AGKE protocols have been defined to formalize the possible threats and attacks.

Paper “*Automating risk analysis of software design models*” by M. Frydman et al. describes an approach to reduce the need for costly human expertise to perform risk analysis in software, which is common in secure development methodologies, by automating threat modeling. Reducing the dependency on security experts aims at reducing the cost of secure development by allowing non-security-aware developers to apply secure development with little to no additional cost, making secure development more accessible.

The paper entitled “*A Regev-type fully homomorphic encryption scheme using modulus switching*” by Z. Chen et al. recommends concrete parameter values of our proposed scheme and provide security analysis. The result shows that the modified FHE scheme is more efficient than the original Brakerski scheme in the same security level.

The paper entitled “*Countermeasures to avoid noncooperation in fully self-organized VANETs*” by J. Molina-Gil et al. proposes a new vehicular communication system based on mobile phones for fully distributed and decentralized networks. In these networks, communications depend on individual nodes, which could decrease the efficiency and reliability of transmitted information.

The paper entitled “*Calculating the number of cluster heads based on the rate-distortion function in wireless sensor networks*” by M. Yang et al. proposes a method for the calculation of the number of cluster heads based on the rate-distortion function after establishing an energy consumption model according to the data fusion framework in WSNs.

The paper entitled “*A game-theoretic response strategy for coordinator attack in wireless sensor networks*” by J. Liu et al. proposes an adaptive coordinator selection algorithm using game and fuzzy logic aiming at both minimizing the average number of hops and maximizing network lifetime. The proposed game model consists of two interrelated formulations: a stochastic game for dynamic defense and a best response policy using evolutionary game formulation for coordinator selection.

The paper “*A model based security testing method for protocol implementation*” by Y. L. Fu and X. L. Xin proposes an extended model of IOLTS to describe the legal roles and intruders of security protocol implementations and then combine them together to generate the suitable test cases to verify the security of protocol implementation. The proposed model inherits the clarity of finite automata and can describe the security properties and most of the protocol behaviors with the definition of transition.

The paper entitled “*A comprehensive review on adaptability of network forensics frameworks for mobile cloud computing*” by S. Khan et al. discussed the functions, approaches, and structures of current NFFs. The authors conclude that new research roadmaps and programs are required to overcome the issues and challenges faced by CSPs. Standardized rules, secure reference models, protocols, trust architectures, legal contemplation, technological development, and a global regularity body should be established. These requirements can be achieved by harmonizing the efforts of industrial experts, academic researchers, and investigators under legal entity bodies.

The aim of the paper entitled “*Combining digital watermarking and fingerprinting techniques to identify copyrights for color images*” by S.-L. Hsieh et al. is to present a copyright identification scheme for color images that takes advantage of the complementary nature of watermarking and fingerprinting. The experimental results showed that when the watermarked image suffers moderate attacks,* watermarking verification* alone is enough to identify the copyright and there is no need for* fingerprinting verification*.

The paper entitled “*Towards dynamic remote data auditing in computational clouds*” by M. Sookhak et al. proposes an effectual RDA technique based on algebraic signature properties for cloud storage system and also presents a new data structure capable of efficiently supporting dynamic data operations like append, insert, modify, and delete. The comparative analysis with the state-of-the-art RDA schemes shows that the proposed scheme is secure and highly efficient in terms of the computation and communication overhead on the auditor and server.

The paper entitled “*Fuzzy-based trust prediction model for routing in WSNs*” by X. Anita et al. proposed FTPR protocol to effectively thwart black hole attack, on-off attack, conflicting behavior attack, and bad-mouthing attack. It employed a fuzzy-based trust prediction model to predict the future behavior of a neighboring node based on its historical behavior, trust fluctuations, and recommendation inconsistency.

In the paper entitled “*Modeling the propagation of mobile phone virus under complex network*” by W. Yang et al., the M-SIS and M-SIR propagation models for mobile phone viruses are proposed, combining with the structural characteristics of the complex network. The M-SIR propagation model is suitable to describe the vulnerability-exploiting mobile phone virus. It reflects the characteristic of the mobile virus, which spreads by exploiting vulnerabilities, and the mobile phone can be immune to the mobile phone virus after virus removal and patching.

The paper entitled “*Malware analysis using visualized image matrices*” by K. S. Han et al. proposes a novel malware visual analysis method that contains not only a visualization method to convert binary files into images, but also a similarity calculation method between these images. The proposed method generates RGB-colored pixels on image matrices using the opcode sequences extracted from malware samples and calculates the similarities for the image matrices.

The paper entitled “*Quality of protection evaluation of security mechanisms*” by B. Ksiezopolski et al. proposes a model for QoP evaluation of security mechanisms. Owing to this model, one can quantify the influence of particular security mechanisms on ensuring security attributes. An additional contribution of the paper is the implementation of the security mechanisms evaluation tool (SMETool) which supports the presented method.

A novel approach described to aid breast cancer detection and classification using digital mammograms is presented by X.-S. Zhang in his paper entitled “*A new approach for clustered MCs classification with sparse features learning and TWSVM*.” The proposed method is based on sparse feature learning and representation, which expresses a testing sample as a linear combination of the built vocabulary (training samples).

The paper entitled “*An action-based fine-grained access control mechanism for structured documents and its application*” by M. Su et al. presents an action-based fine-grained access control mechanism for structured documents. By defining the objective describing model, it could support the permission management on chapters, pages, sections, words, and pictures of structured documents.

The paper entitled “*SmartMal: a service-oriented behavioral malware detection framework for mobile devices*” by C. Wang et al. presents SmartMal—a novel service-oriented behavioral malware detection framework for vehicular and mobile devices. The proposed framework relies on client-server architecture, the client continuously extracts various features and transfers them to the server and the server's main task is to detect anomalies using state-of-art detection algorithms.

In the paper entitled “*A new gravitational particle swarm optimization algorithm for the solution of economic emission dispatch in wind-thermal power system*” by S. Jiang et al., a new hybrid optimization approach, namely, gravitational particle swarm optimization algorithm (GPSOA), is proposed to solve economic emission dispatch (EED) problem including wind power.

The paper entitled “*An analysis of security system for intrusion in smartphone environment*” by M. Louk et al. uses Android OS because the operating system is frequently attacked by cybercriminals. Monitoring, detecting, tracking, and notification are used not only to check new application before being installed into the smartphone, but also to detect suspicious behavior activity in real time.

The paper entitled “*Spatiotemporal access model based on reputation for the sensing layer of the IoT*” by Y. Guo et al. proposes a model that combines space and time with reputation to control access to the information within the sensing layer of the IoT. This model is called spatiotemporal access control based on reputation (STRAC).

The paper entitled “*Security enhanced anonymous multiserver authenticated key agreement scheme using smart cards and biometrics*” by Y. Choi et al. proposes a security enhanced anonymous multiserver authenticated key agreement scheme which addresses all the weaknesses identified in Chuang and Chen's scheme.

The paper entitled “*Self-adaptive trust based ABR protocol for MANETs using Q-learning*” by A. V. Kumar et al. focuses on computing a score using Q-learning to weigh the trust of a particular node over associativity based routing (ABR) protocol.

The paper entitled “*Efficient and privacy-preserving metering protocols for smart grid systems*” by H. J. Jo and D. H. Lee proposes two protocols. The first protocol is based on the signcryption algorithm and achieves weak confidentiality; it is robust against node compromise attacks. The second one is an extended version of the first one; it satisfies strong confidentiality by using the Paillier homomorphic encryption algorithm.

